# Androgen-Influenced Polarization of Activin A-Producing Macrophages Accompanies Post-pyelonephritic Renal Scarring

**DOI:** 10.3389/fimmu.2020.01641

**Published:** 2020-07-28

**Authors:** Teri N. Hreha, Christina A. Collins, Allyssa L. Daugherty, Jessie M. Griffith, Keith A. Hruska, David A. Hunstad

**Affiliations:** ^1^Department of Pediatrics, Washington University School of Medicine, St. Louis, MO, United States; ^2^Department of Cell Biology and Physiology, Washington University School of Medicine, St. Louis, MO, United States; ^3^Department of Molecular Microbiology, Washington University School of Medicine, St. Louis, MO, United States

**Keywords:** urinary tract infection, activin A, follistatin, macrophage polarization, *Escherichia coli*

## Abstract

Ascending bacterial pyelonephritis, a form of urinary tract infection (UTI) that can result in hospitalization, sepsis, and other complications, occurs in ~250,000 US patients annually; uropathogenic *Escherichia coli* (UPEC) cause a large majority of these infections. Although UTIs are primarily a disease of women, acute pyelonephritis in males is associated with increased mortality and morbidity, including renal scarring, and end-stage renal disease. Preclinical models of UTI have only recently allowed investigation of sex and sex-hormone effects on pathogenesis. We previously demonstrated that renal scarring after experimental UPEC pyelonephritis is augmented by androgen exposure; testosterone exposure increases both the severity of pyelonephritis and the degree of renal scarring in both male and female mice. Activin A is an important driver of scarring in non-infectious renal injury, as well as a mediator of macrophage polarization. In this work, we investigated how androgen exposure influences immune cell recruitment to the UPEC-infected kidney and how cell-specific activin A production affects post-pyelonephritic scar formation. Compared with vehicle-treated females, androgenized mice exhibited reduced bacterial clearance from the kidney, despite robust myeloid cell recruitment that continued to increase as infection progressed. Infected kidneys from androgenized mice harbored more alternatively activated (M2) macrophages than vehicle-treated mice, reflecting an earlier shift from a pro-inflammatory (M1) phenotype. Androgen exposure also led to a sharp increase in activin A-producing myeloid cells in the infected kidney, as well as decreased levels of follistatin (which normally antagonizes activin action). As a result, infection in androgenized mice featured prolonged polarization of macrophages toward a pro-fibrotic M2a phenotype, accompanied by an increase in M2a-associated cytokines. These data indicate that androgen enhancement of UTI severity and resulting scar formation is related to augmented local activin A production and corresponding promotion of M2a macrophage polarization.

## Introduction

Urinary tract infections (UTIs) are extremely common, affecting millions of people worldwide. Uropathogenic strains of *Escherichia coli* (UPEC) cause over 80% of UTIs, including both bladder infections (cystitis) and ascending infection of the kidneys (pyelonephritis). UTIs predominantly affect females, though infant and elderly males exhibit higher rates of UTI compared to similarly aged females (1–6). Males also exhibit higher morbidity and mortality than females in the setting of complicated UTI (4, 7). Upper-tract UTI in childhood carries risk for renal scarring, which in turn correlates with risk of chronic kidney disease, and end-stage renal disease later in life (8–14). Our prior studies in mice demonstrated enhanced UTI severity and scar formation in males compared with females, phenotypes shown to be dependent on androgen exposure (15, 16).

Macrophage recruitment, polarization, and function are important for the proper resolution of many bacterial infections. In a typical response, circulating monocytes are recruited to the site of infection upon signaling by damage-associated and pathogen-associated molecular patterns (DAMPs and PAMPs), and proinflammatory cytokines such as IL-6, IFNγ, and TNFα; these arriving monocytes initially differentiate, or polarize, toward proinflammatory (M1) macrophages (17–25). These M1 cells further secrete proinflammatory cytokines and chemokines, exert phagocytic activity, and induce neutrophil apoptosis (25–30). Reduction of local DAMP and PAMP quantities, along with an increase in neutrophil debris, and accumulation of T_H_2 cytokines, including cytokines such as CXCL1, G-CSF, and IL-10 (27, 31–33), subsequently encourages these M1 macrophages to polarize toward alternatively activated M2 macrophages (34–38). M2a macrophages are activated by IL-4 and IL-13, and are considered pro-fibrotic (39–42). These cells secrete TGFβ1 and are involved in cell growth, repair, and matrix deposition. Immune complexes and IL-1β stimulate M2b polarization, which is involved in regulation of the immune and inflammatory responses (43, 44). M2c macrophages are stimulated by IL-10, are involved in phagocytosis and matrix remodeling, and typically signal resolution of the inflammatory response to an injury (45–49).

Activin A, a TGFβ superfamily member that is a homodimer of inhibin β_A_, has been shown to be upregulated in several different systemic infection or injury models (50–56). In models of non-infectious renal injury, activin A signaling promotes renal scarring and fibrosis (55–59); in other systems, activin A has been shown to exert varying effects on macrophage polarization. For example, it encourages an M1 phenotype on unstimulated monocytes and macrophages *in vitro* (60–63) but pushes these cells toward a M2 polarization state when they are primed with LPS (64–68).

Testosterone signaling increases susceptibility to, and severity of, experimental pyelonephritis and renal scars in both male and female mice (69), while anti-androgen treatments are protective against UTI in mice and in women with polycystic ovary syndrome (16, 70, 71). Sex differences are also evident in the immune response to infection, and vary somewhat by model. Males tend to have more circulating M1 macrophages during infection (72), and dihydrotestosterone (DHT) can induce a prolonged M1 macrophage polarization state *in vitro* (73). Females typically exhibit more intense inflammatory responses to multiple microbial stimuli (including vaccines), and have more efficient phagocytic macrophages and increased levels of Toll-like receptors (TLRs) and pro-inflammatory cytokines (74, 75). In contrast, women taking oral contraceptives demonstrated a decrease in several pro-inflammatory cytokines (IFNγ, TNFα) after LPS stimulation (75), and testosterone stimulation has been shown to decrease the production of TLR4 in mice (76).

In mouse models of non-infectious renal injury, aberrant wound healing in males is characterized by increased leukocyte infiltrate and enhanced proteolysis of ECM, while castration promotes favorable wound healing (77, 78). Renal fibrosis in these models is also strongly associated with the presence of M2 macrophages (79–83); in fact, adoptive transfer of M2 macrophages after unilateral ureteral obstruction (UUO) promoted the accumulation of αSMA+ cells (indicative of fibrotic scarring), a phenotype that involved signaling by members of the TGFβ superfamily (84).

Here, we used C57BL/6 females treated with testosterone cypionate (TC) in order to investigate how activin A influences macrophage polarization during ascending pyelonephritis in the androgenized host. Although several studies have investigated how activin A affects macrophage polarization *in vitro* in the presence of LPS, data are sharply lacking on how these interactions transpire during *in vivo* infection. We determined that during ascending UPEC pyelonephritis, androgen exposure results in increased local activin A and promotes recruitment of activin A-producing leukocytes, particularly activin A+ monocytes and macrophages. Further, androgenized mice exhibited decreased local IFNγ and TNFα along with increased CXCL1 and G-CSF, associated with decreased local M1:M2 macrophage ratios throughout infection. In particular, androgen exposure caused a persistent increase in pro-fibrotic M2a macrophages during later stages of infection. This androgen-dependent skewing toward M2a macrophages promotes an environment of reduced bacterial clearance and enhanced renal scarring.

## Materials and Methods

### Bacterial Strains

UTI89, a clinical cystitis isolate of uropathogenic *Escherichia coli* (UPEC) (85), was grown statically overnight in Luria-Bertani broth (LB; Becton Dickinson, Sparks, MD) at 37°C. Overnight cultures were centrifuged for 10 min at 7,500 × *g* at 4°C before resuspension in sterile phosphate-buffered saline (PBS) to a final density of ~4 × 10^8^ colony-forming units (CFU)/mL.

### Animals

All animal protocols received prior approval from the Washington University Institutional Animal Care and Use Committee. Experiments were conducted in female C57BL/6 mice (#000664; Jackson Laboratories, Bar Harbor, ME) or, for immunofluorescence analysis, in female bigenic Gli1-tdTomato^+^ mice, which harbor a tamoxifen-inducible Cre for tdTomato production from the Gli1 promoter [kind gift of B. Humphreys; (86)]. For androgenization, mice of either strain were given weekly intramuscular injections of 150 mg/kg testosterone cypionate (TC, Depo-Testosterone; Pfizer, New York, NY) beginning at 5 wk of age, and continuing until sacrifice. UTI was initiated by inoculation of the bladder with 1–2 × 10^7^ CFU of UPEC *via* catheter at 7 wk of age, as described previously (87, 88).

### Determination of Bacterial Loads

At the indicated time points, mice were anesthetized with inhaled isoflurane (Patterson Veterinary, Greeley, CO) and terminally perfused with 4°C PBS through the left ventricle. Bladders and kidneys were aseptically removed and homogenized in 4°C PBS. The resulting tissue homogenates were serially diluted and plated on LB agar.

### Tissue Preparation and Histology

Gli1-tdTomato^+^ Mice were euthanized as described above, and aseptically removed kidneys were fixed in 4% paraformaldehyde in PBS for 1 h at 4°C, incubated overnight in 30% sucrose in PBS at 4°C, then embedded in OCT (Fisher Scientific, Hampton, NH). Embedded kidneys were cryosectioned into 5–8-μm sections and mounted onto Superfrost Plus slides (Fisher Scientific). For immunofluorescence staining, sections were washed with PBS, blocked with 10% fetal bovine serum (FBS) in PBS, then stained with fluorescently conjugated primary antibodies against CD206-Alexa Fluor 488 (1:200; Biolegend #141709) and CD80-APC (1:200; Biolegend #104713). Sections were then washed with PBS, stained with 1:5,000 4′,6-diamidino-2-phenylindole (DAPI) and mounted with ProLong Gold (both from Life Technologies, Carlsbad, CA). Images were captured digitally with a Zeiss LSM 880 Airyscan confocal microscope (Oberkochen, Germany).

### Flow Cytometry

Kidneys were harvested as described above, and were manually homogenized into cold RPMI (Gibco) before treatment with RBC lysis buffer (155 mM NH_4_Cl, 10 mM KHCO_3_) at room temperature to ensure complete lysis of any remaining RBCs. After washing, cells were subjected to a Percoll gradient (Percoll PLUS; GE Healthcare, Uppsala, Sweden) in FACS buffer [10% FBS, 1% w/v sodium azide, 2 mM ethylenediaminetetraacetic acid (EDTA) in PBS] + 25 mM sucrose for leukocyte enrichment, then resuspended in 4°C PBS and stained with Live/Dead Fixable Yellow (ThermoFisher Scientific). Cells were washed again, resuspended in 4°C FACS buffer and blocked with Fc Block (BD Biosciences, San Jose, CA) on ice, followed by staining with fluorescently conjugated antibodies against the following extracellular antigens: CD45-BV510 (1:200; BD Biosciences #563891), NK1.1-AlexaFluor 700 (1:50; Biolegend #108730, San Diego, CA), CD11c-AlexaFluor 700 (1:200; Biolegend #117320), Ly6G-AlexaFluor 700 (1:200; Biolegend #127621), CD19-AlexaFluor 700 (1:200; Biolegend #115527), CD3e-AlexaFluor 700 (1:100; BD Biosciences #557984), CD150-APC (1:100; Biolegend #115910), CD206-PE-Cy7 (1:100; Biolegend #141719), CD86-PE-Cy5 (1:100; Biolegend #105016), CD115-PE (1:100; Biolegend #135506), CD80-FITC (1:50; Biolegend #104706). After staining, cells were washed, fixed in 4% paraformaldehyde in PBS, permeabilized on ice with Perm/Wash buffer (10% FBS, 1% w/v sodium azide, 1.3 mM saponin in PBS, pH 7.4–7.6), and then stained with the intracellular antibody Inhibin β_A_-MaxLight405 (1:20; US Biological #211496, Salem, MA). All macrophages described are CD11b+ and Ly6C+. M1 macrophages are defined as CD80+, F4/80+, MHC-II lo. M2a macrophages are defined as CD206+ F4/80+ and MHC-II lo/−; M2b as CD86+, F4/80+/−, MHC-II lo/−; and M2c as CD150+, F4/80+/−, MHC-II hi (data not shown). For flow cytometry of whole-kidney activin A production, the kidneys were processed as described above, but cell suspensions were not subjected to the Percoll gradient. After blocking, cells were stained with labeled antibodies against the following extracellular antigens: E-cadherin (CD324)-PE-Cy7 (1:200, Biolegend #147309), and CD45 (30-F11)-BV510 (1:200, BD Biosciences #563891) and the intracellular antibody Inhibin β_A_-MaxLight405 (1:20; US Biological #211496) as described above. Stained cells were washed, resuspended in FACS buffer and subjected to flow cytometry on a LSR II Fortessa instrument (BD Biosciences). Results were analyzed using FlowJo software (BD Biosciences). A representative gating scheme is provided in Figure S1.

### Immunoblotting

Harvested kidneys were flash frozen in liquid nitrogen and stored at −80°C until use. Kidneys were homogenized in RIPA buffer (50 mM Tris-HCl, 150 mM NaCl, 1% v/v Nonidet P-40, 0.1% w/v SDS, 0.5% w/v sodium deoxycholate, pH 7.4) containing PhosSTOP phosphatase inhibitor (Roche; Basel, Switzerland) and complete Mini protease inhibitor (Roche). The lysates were cleared by centrifugation (2 × 5 min at max speed in a tabletop centrifuge), followed by total protein quantification by BCA assay (Invitrogen, Carlsbad, CA). Eighty μg of protein was run on SDS-PAGE gels and transferred to PVDF membranes. Membranes were blocked with 5% w/v non-fat milk (Carnation, Vaud, Switzerland) in PBS containing 0.05% v/v Tween-20 (PBST), and probed with primary antibodies against follistatin (1:500; Invitrogen # PA5-79284) and CoxIV (1:20,000; Cell Signaling Technologies #4844, Danvers, MA) in blocking buffer overnight at 4°C. Membranes were washed and probed 1:2,000 with the appropriate horseradish peroxidase-conjugated secondary antibody (GE Healthcare #NAP34) in blocking buffer for 1 h at room temperature. Membranes were washed again and developed with the Clarity Western ECL Kit (Bio-Rad, Hercules, CA).

### Cytokine Quantification

Protein was extracted from flash-frozen kidneys as described above, and diluted in PBS to 900 μg/mL. The diluted protein was analyzed with a customized Bio-Plex Pro Mouse Cytokine Group I kit (Bio-Rad) according to the manufacturer's instructions. The plate was read with a Bio-Plex 200 system and analyzed using BioPlex Manager 6.1 software.

### qPCR

mRNA was extracted from flash-frozen kidneys using RNA Stat-60 (amsbio, Cambridge, MA) according to package instructions. One μg mRNA was converted to cDNA using the iScript cDNA Synthesis Kit (Bio-Rad) according to package instructions. qPCR was performed with the SsoAdvanced Universal SYBR Green Supermix (Bio-Rad), containing ~20 ng of cDNA and 350 nM primers. Thermal cycling was performed on a 7500 Fast RT-PCR system (Applied Biosystems, Foster City, CA) with the following protocol: 95°C, 3 min; 40 × (95°C, 10 s; 60°C, 30 s). A list of primer sequences is provided in Table S1.

### Statistical Analysis

Statistical analysis for CFU and Bio-Plex data was performed using the non-parametric Mann-Whitney U-test. All other statistics were performed with an unpaired *t*-test. *P* < 0.05 were considered significant.

## Results

### Androgen Exposure Amplifies Renal Activin Expression During Pyelonephritis

**Figure 1:**
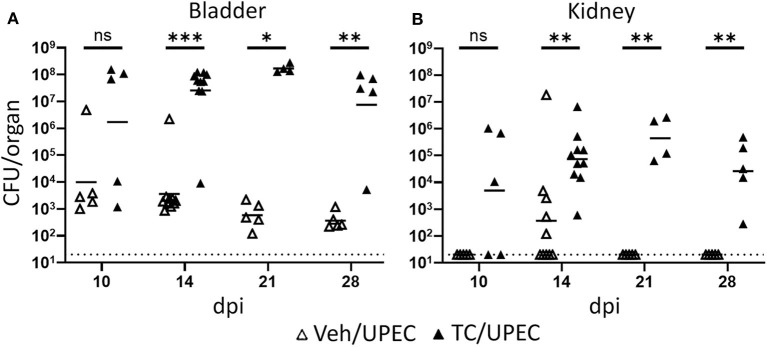
Androgenized mice exhibit severe UTI. Organ titers (CFU) were quantified in serially diluted bladder **(A)** or kidney **(B)** homogenates at the indicated time points post UPEC infection of vehicle-treated mice (open triangles) or TC-treated mice (filled triangles). Dotted line indicates the limit of detection; dpi, days post-infection. *n* = 4–10 mice per group. *P < 0.05, **P < 0.01, ***P < 0.001.

**Figure 2:**
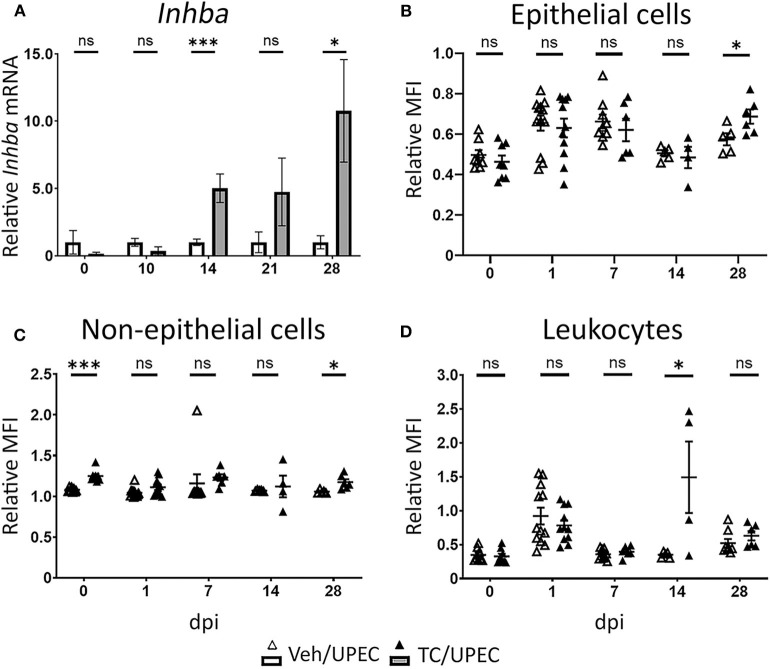
Activin A expression and production is increased in the kidneys of androgenized mice. **(A)** Relative whole-kidney mRNA expression of *Inhba* was determined in vehicle-treated mice (open bars) and TC-treated mice (filled bars) by qPCR at various time points post UPEC infection. *n* = 4–8 mice per group. The relative mean fluorescence intensity (MFI) of activin A in **(B)** epithelial cells (CD45− E-cadherin+), **(C)** non-epithelial cells (CD45− E-cadherin−), or **(D)** leukocytes (CD45+ E-cadherin−) compared to the MFI in the total live cell population was determined by flow cytometry at the indicated time points in vehicle-treated mice (open triangles) or TC-treated mice (filled triangles). *n* = 4–10 mice per group. *P < 0.05, ***P < 0.001.

In agreement with our previous work (16, 69), TC-treated (androgenized) mice maintained consistently high UPEC titers in both bladders and kidneys, significantly higher than those in vehicle-treated mice beginning 14 days post infection (dpi; Figure 1). As infection progressed, kidneys of TC-treated mice had increased global transcription of *Inhba* (encoding activin A) beginning 14 dpi and continuing through 28 dpi (Figure 2A). This increased transcription led to modest but statistically significant increases in activin A production 28 dpi by both epithelial (CD45– E-cadherin+; Figure 2B) and non-epithelial cells (CD45– E-cadherin–; Figure 2C), as determined by flow cytometry. This increase in activin A is consistent with similar increases seen in other renal injury models (55, 56). Meanwhile, the leukocyte (CD45+) population in TC-treated mice showed a significant elevation of activin A production 14 dpi (Figure 2D). This activin burst was of much greater amplitude than that seen in the other cell populations, leading us to investigate further how activin production by leukocyte populations could associate with the reduced UPEC clearance and enhanced scar formation seen in the androgenized host.

### Follistatin Production Is Suppressed in Androgen-Exposed Mice With UTI

**Figure 3:**
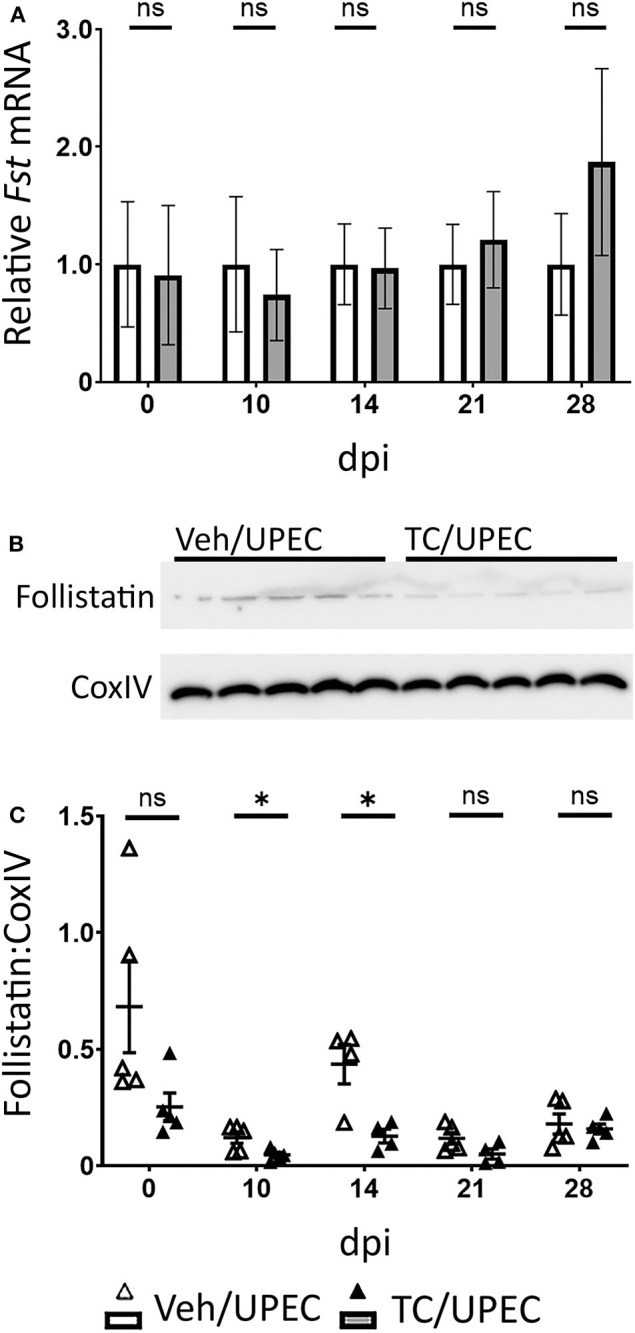
Follistatin production is reduced during pyelonephritis in androgenized mice. **(A)** Relative whole-kidney *Fst* mRNA was measured by qPCR at the indicated time points in vehicle-treated mice (open bars) and TC-treated mice (filled bars). *n* = 4–8 mice per group. Whole-kidney protein production of follistatin was determined by quantitative western blot [representative blot shown in **(B)**; quantitation in **(C)**] at the indicated time points in vehicle-treated mice (open triangles) and TC-treated mice (filled triangles). *n* = 4–5 mice per group. *P < 0.05.

Follistatin binds strongly to activin A in the circulation and tissues, preventing its binding to its cellular receptor and thereby rendering it inactive (89–91). We hypothesized that renal tubular epithelial cell death associated with UPEC infection would reduce local production of follistatin (16). Indeed, while whole-kidney transcription of follistatin during UPEC infection was not altered in TC-treated mice (Figure 3A), follistatin production in whole-kidney homogenates was significantly reduced in TC-treated mice 10 and 14 dpi, as measured by quantitative immunoblot (Figures 3B,C). There was mild (but not statistically significant) reduction in follistatin production in androgenized mice across the other sampled time points (Figure 3C). Taken together, increased activin A production, coupled with decreased follistatin production, would provide an environment in the androgenized mouse kidney with increased activin A activity during UPEC infection.

### Androgenized Mice Harbor Increased Activin A-Producing Myeloid Cells in the Infected Kidney

**Figure 4:**
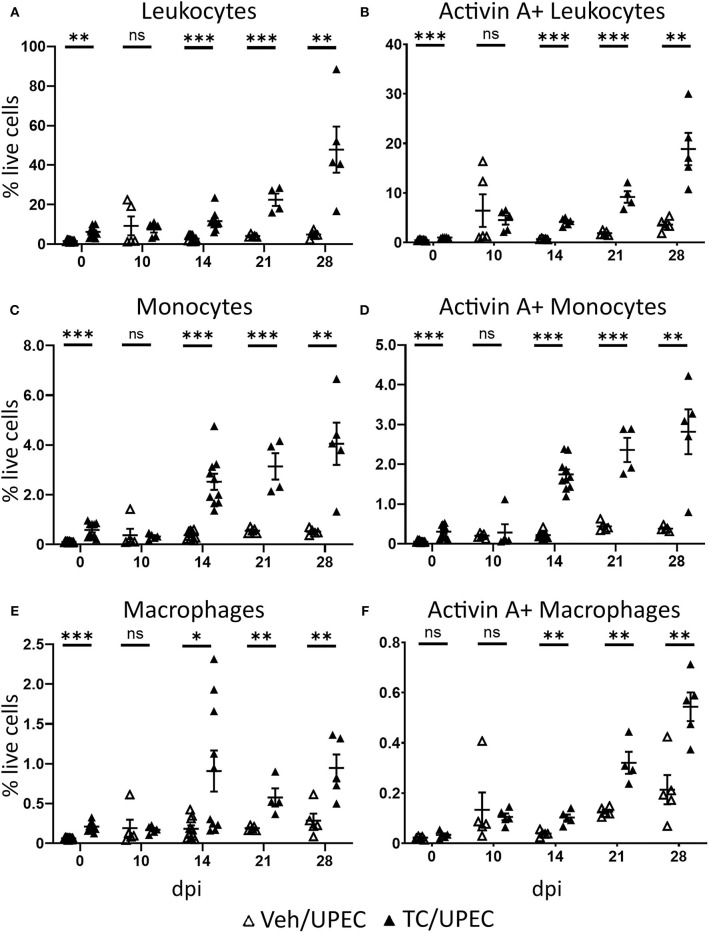
Androgenized mice have larger populations of activin A-producing leukocytes, including monocytes and macrophages, in the kidneys during UPEC infection. The population of **(A)** total leukocytes (CD45+), **(B)** activin A+ leukocytes, **(C)** monocytes (CD45+ CD115+ CD19− CD3e− Ly6G− CD11c− NK1.1−), **(D)** activin A+ monocytes, **(E)** macrophages (CD45+ CD115− CD19− CD3e− Ly6G− CD11c− NK1.1−), and **(F)** activin A+ macrophages as a percentage of the total live cell population was determined by flow cytometry in vehicle-treated mice (open triangles) and TC-treated mice (filled triangles) at the indicated time points. *n* = 4–10 mice per group. *P < 0.05, **P < 0.01, ***P < 0.001.

Activin A has been shown to affect macrophage polarization *in vitro*, encouraging M1 polarization in unstimulated macrophages while promoting M2 polarization in LPS-stimulated models (56–64). We examined leukocyte (CD45+) populations within the kidneys of TC-treated mice at various time points in order to interrogate the role of androgens in activin A-driven macrophage polarization during pyelonephritis. After 14 dpi, TC-treated mice consistently exhibited increased recruitment of CD45+ cells to the kidneys compared to vehicle-treated mice (Figure 4A). While most of these CD45+ cells were neutrophils (Ly6G+; data not shown), TC-treated mice displayed a sustained increase in both monocyte (CD19− CD3e− Ly6G− CD11c− NK1.1− CD115+) and macrophage (CD19− CD3e− Ly6G− CD11c− NK1.1− CD115−) populations in the kidneys starting 14 dpi (Figures 4C,E). There were also more activin A+ leukocytes, monocytes, and macrophages in the kidneys of androgenized mice, indicating that both the monocyte and macrophage populations were contributing to activin A signaling in the infected kidney (Figures 4B,D,F).

### Androgen Exposure Favors Polarization of Renal Macrophages Toward the Pro-fibrotic M2a Phenotype

**Figure 5:**
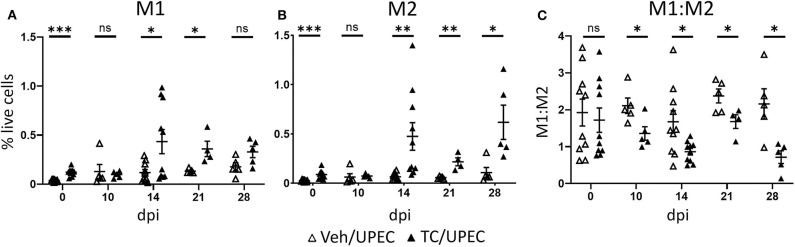
Androgenized mice have increased populations of both M1 and M2 macrophages, but a reduced M1:M2 ratio. The populations of **(A)** M1 macrophages (CD80+) and **(B)** M2 macrophages (CD80−) as a percentage of total live cells was determined by flow cytometry at the indicated time points in vehicle-treated mice (open triangles) and TC-treated mice (filled triangles). **(C)** The ratio of M1 to M2 macrophages for each mouse was calculated from the data represented in **(A,B)**. *n* = 4–10 mice per group. *P < 0.05, **P < 0.01, ***P < 0.001.

To investigate how the increased levels of activin A affected macrophage polarization during UPEC infection and resolution, we quantified kidney macrophages in the M1 or M2 polarization states at various time points. Compared with vehicle-treated mice, androgenized mice harbored an increased population of M1 macrophages (CD80+; Figure 5A) in the kidneys 14 and 21 dpi, and an even greater increase in M2 macrophages from 14 to 28 dpi (CD80−; Figure 5B). This led to an overall decrease in the M1:M2 ratio, beginning 10 dpi and sustained throughout the course of infection (Figure 5C). A prolonged reduction in the M1:M2 ratio is reflective of aberrant wound healing and is associated with fibrotic scarring (25).

**Figure 6:**
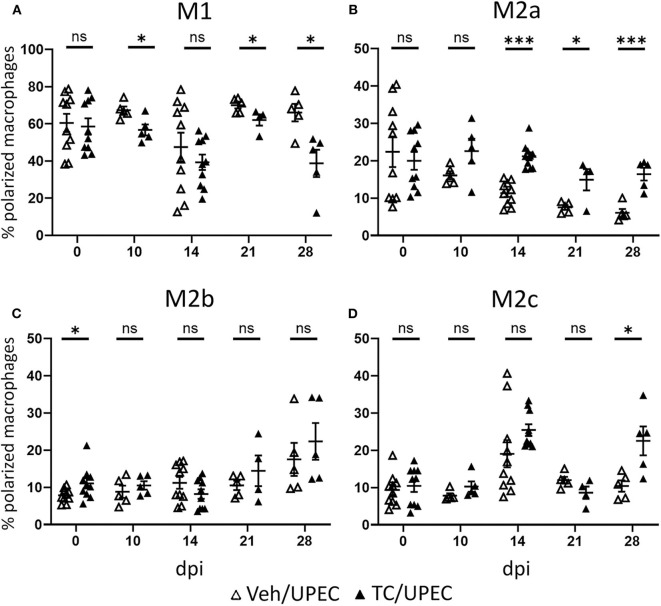
Androgenized mice harbor an increased proportion of M2a polarized macrophages. The population of **(A)** M1 macrophages (CD80+), **(B)** M2a macrophages (CD80− CD206+ CD150−), **(C)** M2b macrophages (CD80− CD86+), and **(D)** M2c macrophages (CD80− CD150+) as a percentage of the polarized macrophage population was determined by flow cytometry at various time points in vehicle-treated mice (open triangles) and TC-treated mice (filled triangles). *n* = 4–10 mice per group. *P < 0.05, ***P < 0.001.

Within the population of activated macrophages, the M1 phenotype predominated in both vehicle and TC-treated mice throughout the course of infection; however, androgenized mice showed a significant reduction at multiple time points in the fraction of polarized macrophages that were M1 (Figure 6A). Correspondingly, androgenized mice exhibited a significant increase in M2a (CD206+, CD150−) macrophages, beginning 14 dpi and persisting through the remainder of the course (Figure 6B). Both M1 and M2a macrophages were visualized near populations of Gli1+ activated myofibroblasts, which are the major producers of extracellular matrix proteins in fibrotic injury (Figure S2) (86, 92). Vehicle- and TC-treated mice showed equivalent increases in M2b (CD86+) macrophages at later time points following infection (Figure 6C). Vehicle- and TC-treated mice also harbored similar proportions of M2c (CD150+) macrophages in the kidneys until 28 dpi, when androgenized mice had significantly more (Figure 6D). These results indicate that androgens promote activin A production by myeloid cells responding to UPEC pyelonephritis, with a corresponding increase in M2a polarization of renal macrophages.

### Androgens Promote M2a-Associated Cytokine Expression During Pyelonephritis

**Figure 7:**
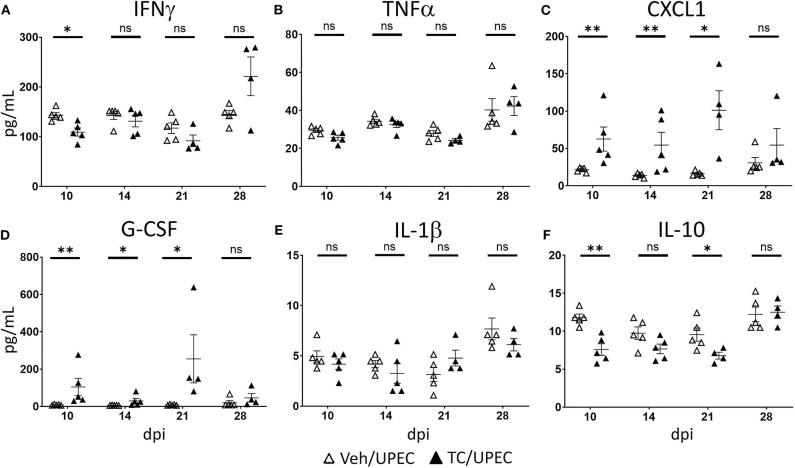
Kidneys of androgenized mice contain reduced M1- and increased M2-polarizing cytokines. The concentrations of **(A)** IFNγ, **(B)** TNFα, **(C)** CXCL1, **(D)** G-CSF, **(E)** IL-1β, and **(F)** IL-10 were quantified by Bio-Plex assay from protein extracted from whole kidneys of vehicle-treated mice (open triangles) and TC-treated mice (filled triangles) at the indicated time points post-infection. *n* = 4–10 mice per group. *P < 0.05, **P < 0.01.

M2a macrophages have been associated with tissue fibrosis after non-infectious injury (39, 40, 93, 94). These cells secrete a number of cytokines and chemokines involved in immunomodulation and repair, including TGFβ1, a chief signaling factor in renal fibrosis (84, 95, 96). Further, adoptive transfer of M2a macrophages led to reduced healing and increased fibrosis of endometriotic lesions (97). We investigated cytokine content in the kidneys of vehicle and TC-treated mice throughout infection. Notably, among M1-associated cytokines, IFNγ was significantly reduced in androgenized mouse kidneys 10 dpi (Figure 7A), while TNFα was unaltered by androgen exposure (Figure 7B). Meanwhile, M2-activating cytokines CXCL1 and G-CSF were significantly increased in TC-treated mice at multiple time points (compared with vehicle-treated; Figures 7C,D), indicating that the cytokine profile of the infected, androgenized kidney may help to drive recruited macrophages toward the M2 polarization state. In line with the flow cytometry data (Figure 6B), TC treatment did not alter the level of M2b stimulant IL-1β in the kidneys (Figure 7E) and acted to depress production of the M2c stimulant IL-10 (Figure 7F). This lack of increase in IL-1β and IL-10 may discourage progression of M2a macrophages toward the M2b and M2c phenotypes that would characterize an optimal healing process.

## Discussion

Our published studies showed that testosterone exposure favors the development of severe pyelonephritis in both C3H and C57BL/6 mice (16, 69), with exacerbation of post-pyelonephritic scarring. The present work demonstrates that androgens encourage a reduction in pro-inflammatory M1 macrophages in the UPEC-infected kidney, conversely favoring the sustained presence of pro-fibrotic M2a macrophages, prolonging UTI and offering a cellular basis for the altered resolution and enhanced scarring we demonstrated previously.

Activin A, a member of the TGFβ superfamily, is involved in both healing and renal fibrosis in several models (55–59) and is a major driver of macrophage polarization (56–64). TC-treated mice demonstrated an increase in *Inhba* transcription and activin A production throughout their kidneys, with a corresponding decrease in follistatin. The cumulative result of these effects is more active activin A in the kidneys of androgen-exposed mice. Interestingly, the CD45+ leukocyte population in TC-treated mice showed the most pronounced increase in activin A (14 dpi); correspondingly, infiltration of multiple myeloid lineages was enhanced in androgenized mice, and the number of activin A-producing cells in these groups also steadily increased.

Activin A signaling has been shown to encourage recruited monocytes to differentiate into either pro-inflammatory M1 macrophages or alternatively activated M2 macrophages (98). This variance in polarization states appears to be environmentally dependent, with unstimulated monocytes and macrophages favoring an M1 phenotype (56–59), while LPS stimulation before activin A treatment skews these cells toward an M2 phenotype (60–64). During active bacterial infection, as in our model, the kidney is exposed to extensive LPS stimulation. This, combined with the increase in activin A, caused androgenized mice to have a sustained preponderance of M2 macrophages. When we examined the specific polarization states of these M2 cells, we found that TC-treated mice harbored significantly more M2a macrophages at all time points beginning 14 dpi. Macrophage polarization and proliferation occurs within the injured kidney, and M2 macrophages are highly important for repair of non-infectious renal injury (99–101). Specifically, M2a macrophages are known to be pro-fibrotic, enhancing TGFβ1 expression, cell growth, tissue repair, and matrix remodeling (39–42). During optimal recovery from tissue injury, this M2a population subsides as they differentiate toward (and are replaced by) immunoregulatory M2b and M2c macrophages, allowing the inflammatory response to abate and the affected tissue to return to a healed state (36, 96, 102, 103). In our model, while M2b and M2c numbers increased slightly over time in both TC- and vehicle-treated mice, the augmented M2a population in androgenized mice did not subside. The persistence of these M2a macrophages would act to prolong the pro-fibrotic state, prevent resolution of inflammation, and favor the androgen-enhanced renal scarring we have shown previously (15, 16).

Macrophage polarization is also highly dependent on secreted cytokines that are secreted by the injured tissue and the macrophages themselves (27, 96). M1 polarization occurs via stimulation with several pro-inflammatory signals (e.g., LPS and IFNγ, with ensuing TNFα, and IL-6 production) (15–23), as are normally elicited early after bacterial infection of the urinary tract (104–106). M2 macrophages are sensitive to a variety of Th2 cytokines, including CXCL1, G-CSF and IL-10 (27, 31–33). The whole-kidney cytokine profiles following UPEC infection aligned with the macrophage polarization states we observed, with androgenized mice exhibiting suppressed IFNγ and unaltered TNFα, accompanied by increased CXCL1 and G-CSF. The depressed IL-10 levels during infection in androgenized mice may hinder the adoption of M2b or M2c phenotypes, restraining kidney macrophages in a prolonged M2a state.

In total, our data indicate that testosterone exposure alters the typical response to renal UPEC infection, pushing the kidney toward a dysfunctional healing process through increased activin A signaling and altered cytokine release. These signals encourage the recruited monocytes to polarize toward and persist as M2a macrophages for weeks in the kidney, preventing bacterial clearance and proper resolution of inflammation. A deeper understanding of how testosterone regulates these signals may allow us to modulate this immune response to help mitigate adverse long-term sequelae of severe pyelonephritis.

## Data Availability Statement

The datasets generated for this study are available on request to the corresponding author.

## Ethics Statement

The animal study was reviewed and approved by the Institutional Animal Care and Use Committee, Washington University School of Medicine.

## Author Contributions

TH, KH, and DH conceived the study. TH, CC, AD, and JG designed and performed experiments. DH and KH critically reviewed the data. TH generated the first manuscript draft. TH, DH, JG, and KH edited the manuscript. All authors contributed to the article and approved the submitted version.

## Conflict of Interest

DH serves on the Board of Directors for BioVersys AG, Basel, Switzerland. The remaining authors declare that the research was conducted in the absence of any commercial or financial relationships that could be construed as a potential conflict of interest.
