# Supplementary material for: Androgen-Influenced Polarization of Activin A-Producing Macrophages Accompanies Post-pyelonephritic Renal Scarring
Source: Front Immunol. 2020 Jul 28;11:1641. doi: 10.3389/fimmu.2020.01641 (PMC7399094; doi:10.3389/fimmu.2020.01641)
Supplement: Supplementary file 1 [file Data_Sheet_1.docx]

*Frontiers in Immunology*

Supplementary information

Androgen-influenced polarization of activin A-producing macrophages accompanies post-pyelonephritic renal scarring

Teri N. Hreha,^1^ Christina A. Collins,^1^ Allyssa L. Daugherty,^1^ Jessie M. Griffith,^1^

Keith A. Hruska,^1,2^ and David A. Hunstad^1,3^*

Departments of ^1^Pediatrics, ^2^Cell Biology and Physiology, and ^3^Molecular Microbiology, Washington University School of Medicine, St. Louis, MO


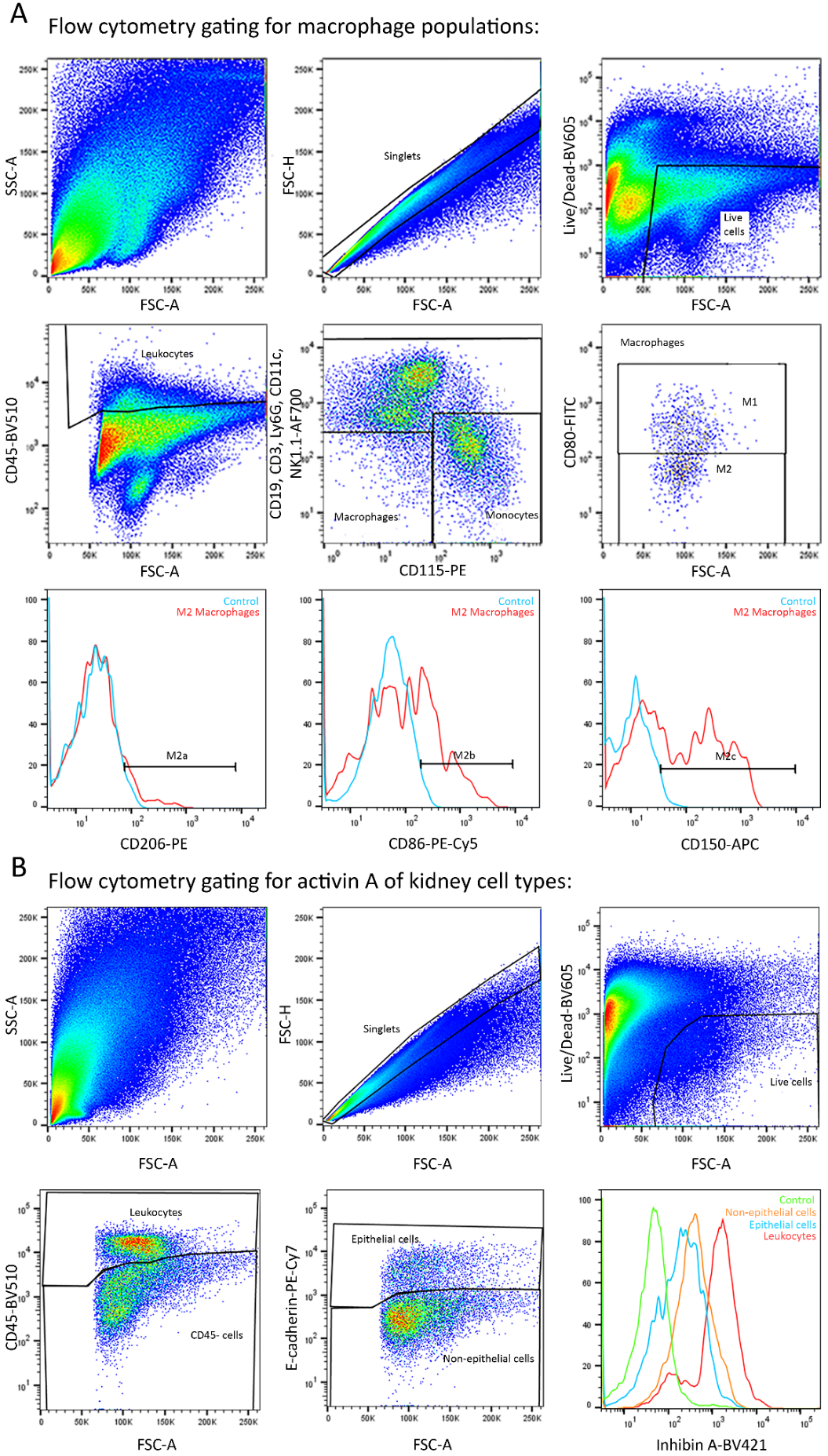


**Figure S1.** Representative gating scheme for the flow cytometric analysis of (A) macrophage populations and (B) kidney cell populations used in this study.


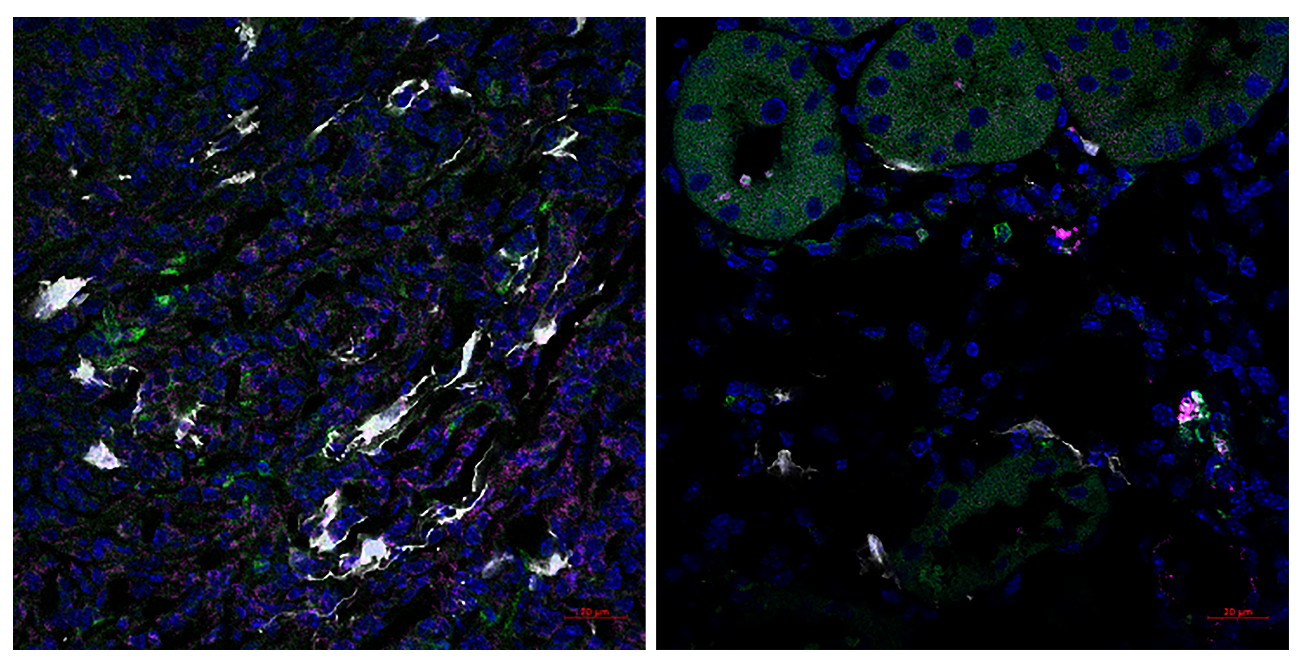


**Figure S2.** CD80+ M1macrophages (fuchsia) and CD206+ M2a (green) macrophages localized near Gli1+ activated myofibroblasts (white) 14 dpi in Gli1-tdTomato^+^ mice. Nuclei are stained with DAPI (blue). Scale bar represents 20 µm.

**Table S1.** Primers used for qPCR

| **Gene** | **Encoded Protein** | **Forward Primer Sequence**  **(5’ 🡪 3’)** | **Reverse Primer Sequence**  **(5’ 🡪 3’)** |
| --- | --- | --- | --- |
| *Gapdh* | Gapdh | TGTTACCAACTGGGACGACA | GGGGTGTTGAAGGTCTCAAA |
| *Inhba* | Activin A | GGAGATAGAGGACGACATTGGC | ACGCTCCACTACTGACAGGTCA |
| *Fst* | Follistatin | GCCAGTGACAATGCCACATACG | CTTCCTCCGTTTCTTCCGAGATG |
